# Electronic hand hygiene monitoring systems are the wave of the future

**DOI:** 10.1017/ash.2022.241

**Published:** 2022-06-06

**Authors:** Joshua K. Schaffzin

**Affiliations:** Department of Pediatrics, University of Cincinnati College of Medicine, Cincinnati, Ohio and Division of Infectious Diseases, Cincinnati Children’s Hospital Medical Center, Cincinnati, Ohio

In the busy, ever-expanding world of infection prevention and control (IP&C), implementing electronic hand hygiene monitoring systems (EHHMS) can help guide focused improvement efforts, maximize adherence, and prevent transmission of nosocomial pathogens.

## Cost

A common concern for adopting an EHHMS is cost. Systems are expensive, and requirements include deployment of sensors (for locations, dispensers, and staff) and amplifiers, as well as analytic support.^1^ However, once installed, if a clinic or unit layout does not change, costs are reduced to maintenance only. Lifetime costs should be considered. For example, analytics are needed in multiple areas of an institution. Hiring an analyst for EHHMS alone may not be financially feasible, but having that person cover other areas (eg, essential IP&C initiatives like ultraviolet disinfection and healthcare-acquired infection [HAI] tracking, preventive and responsive maintenance, ambulatory staffing and flows, inpatient and ambulatory flow, environmental service metrics, billing efficiency, etc) may provide enough value to an institution to justify hiring.^1^ In accounting for up-front costs, implementation should be purposeful, such as matching device placement to existing flows or taking sensor placement into account when designing new or renovated space.^1^ These efforts will benefit the EHHMS but will also provide data to use in process improvement and design that could benefit a clinic or unit for years to come. Systems based on radio frequency identification (RFID) detection can be utilized to optimize staffing and clinical flow.^2,3^ In fact, staff identifications may already have embedded RFID used for parking and door access. All of these efforts can lead to improved satisfaction and cost savings and make compelling arguments for institutional decision makers to approve the start-up costs.

Investment in an EHHMS is not trivial, but neither is the investment in a manual system. Lifetime costs of a manual system are significant, including training for observation, the observations themselves, and data analysis and distribution.^4^ As a low-reliability intervention,^5^ manual monitoring requires constant education and reminders, and inevitable retraining when observers change (eg, unit reassignment, choosing to stop observing, etc). Data need to be entered and cleaned prior to analysis. If we assume that (1) training for trainers and observers requires 4–6 hours, (2) an observer can complete 5–10 observations in a 20–30-minute observation session, and (3) an observer will be expected to conduct at least 200 observations per unit per month, the institution is committing at least 6.7 hours of nonproductive time per observer per month.^6^ These costs accumulate quickly and recur with staffing changes, which can affect hospital staffing and revenue. Healthcare is facing staffing challenges due to the COVID-19 pandemic, burn-out, and shifting priorities among the work force.^7,8^ Hospitals can ill afford to add additional effort not balanced by revenue like manual hand hygiene observations. Furthermore, if IP&C is responsible for HH observation and/or education, IPs will be diverted from other important initiatives that could be achieved with an EHHMS in place.

## Audit and feedback

Manual observation is often praised for the opportunity it provides for immediate interaction between providers and observers. Often termed ‘audit and feedback,’ correction of improper practice along with praise for proper practice have been shown to improve adherence to hand hygiene and other improvement efforts.^9,10^ However, if the feedback is not well received, it can create tension between observers and providers that can affect both morale and practice. Infection preventionists are often bearers of unfavorable news and work to avoid being seen as enforcers rather than supporters. Conducting HH observations and perceived criticism of provider practice could shift the balance unfavorably. Delegating observations to unit staff may not improve the situation if teammates are not comfortable ‘informing’ on their colleagues’ deficiencies.^11^

An EHHMS does not eliminate audit and feedback opportunities. The wide coverage and extensive sampling an EHHMS offer can be used to identify ‘trouble spots’ to target interventions that generate value. The most effective means of improving HH adherence is through targeted multimodal intervention, which has been demonstrated using manual observation or EHHMS.^12,13^ Rather than prohibit audit and feedback, an EHHMS can identify spots where receptiveness to intervention, observation, and direct feedback would be high, and this information can be incorporated with other interventions to generate a desirable outcome.

## Accuracy

EHHMS may be criticized for accuracy, since observation of all 5 World Health Organization moments is not achievable, and some systems infer hand hygiene based on provider presence rather than action.^14,15^ Part of this deficiency can be overcome by sensor placement based on where tasks are likely to take place. For example, if rooms have a designated area for medication preparation, sensors may be able to detect a provider’s presence and whether HH was performed. Similarly, if a urinary catheter collection bag is emptied into a toilet, a provider may be tracked from bed to bathroom. Not all available EHHMSs are able to track activities with high resolution. Nonetheless, technology advancement rarely takes long, and the next generation of EHHMSs promises to provide the details needed.

The accuracy of manual observation can be affected by the Hawthorne effect and observation bias, to which EHHMS are not subject.^16–18^ Even though some studies have shown that the Hawthorne effect can be overcome,^19^ the presence of observers has been shown to improve adherence with HH.^20^ Efforts to minimize the Hawthorne effect, such as ‘secret shoppers,’ require extensive training and maintenance that may not provide much valuable information. When unit staff are recruited to perform manual observations, they often overcompensate for their colleagues, in one study by as much as 20%.^21,22^

Given the need for multimodal intervention, implementing an EHMMS by itself is unlikely to show a significant effect on HAI rates, and stakeholders may question its reliability or cost effectiveness.^23^ The same is true for manual monitoring systems. Like any new initiative, to be successful, groundwork must be done early in the process. This work should include stakeholders in the decision process, testing multiple systems, evaluating usability and acceptability, and developing validation prior to deployment.^24–27^

## The solution?

In the end, neither an EHHMS nor manual observation can fill all system gaps in terms of cost, accuracy, or acceptability. However, while difficult to prove directly, the principle of high HH adherence leading to reduced nosocomial disease is accepted generally. A system chosen by an institution must effectively achieve the goal of reduced transmission. Historically, manual observation has been considered the gold standard for obtaining HH adherence data. Should that continue into the 21st century? The complexity of health care has and will continue to grow rapidly, and technologies have been introduced along the way that have supported providers to achieve positive outcomes. Undetected transmission of bacterial and viral agents is likely occurring in all healthcare contexts, and HH is an essential component of transmission prevention. With evolving technology, widening IP&C responsibilities, and opportunities to incorporate informatics into multiple processes, EHHMS is the future state for HH efforts.
